# A village level cluster-randomized entomological evaluation of combination long-lasting insecticidal nets containing pyrethroid plus PBO synergist in Southern Mali

**DOI:** 10.1186/s12936-017-2124-1

**Published:** 2017-11-21

**Authors:** Moussa B. M. Cisse, Djibril Sangare, Richard M. Oxborough, Abdourhamane Dicko, Dereje Dengela, Aboubacar Sadou, Jules Mihigo, Kristen George, Laura Norris, Christen Fornadel

**Affiliations:** 1 Université des Sciences Techniques et Technologiques de Bamako (USTTB), Bamako, Mali; 2 Malaria Research and Training Center (MRTC), University of Bamako, Bamako, Mali; 3 PMI Africa Indoor Residual Spraying Project, Abt Associates 4550 Montgomery Ave, Suite 800 North, Bethesda, MD 20814 USA; 4 Programme National de Lutte Contre le Paludisme (PNLP), Ministère de la Santé, Bamako, Mali; 5 President’s Malaria Initiative USAID, ACI2000; Rue 243, Porte 297, BP 34, Bamako, Mali; 6 President’s Malaria Initiative USAID, 1300 Pennsylvania Avenue NW, Washington, DC USA

**Keywords:** LLIN, PBO, MFOs, Combination nets, *Anopheles gambiae*, Pyrethroid resistance, Mali, Permanet 3.0, Olyset Plus

## Abstract

**Background:**

There is growing concern that malaria vector resistance to pyrethroid insecticides may reduce the effectiveness of long-lasting insecticidal nets (LLINs). Combination LLINs are designed to control susceptible and pyrethroid-resistant mosquito populations through a mixture of pyrethroid with piperonyl butoxide (PBO) synergist. A cluster randomized trial with entomology outcome measures was conducted in Mali to determine the added benefit over mono-treated pyrethroid predecessors. Four LLIN treatments; permethrin + PBO, permethrin, deltamethrin + PBO, and deltamethrin, were randomly allocated to four villages each (16 villages total) and distributed to cover every sleeping place. Entomological monitoring of indoor *Anopheles* resting densities, host preference, vector longevity, and sporozoite rates were monitored every 2 months over 2 years in 2014 and 2015.

**Results:**

Bottle bioassays confirmed permethrin and deltamethrin resistance in *Anopheles gambiae* sensu lato (s.l.), (the predominant species throughout the study) with pre-exposure to PBO indicating partial involvement of oxidases. Between 2014 and 2015 the mean indoor resting density was greater in the deltamethrin + PBO LLIN arm than the deltamethrin LLIN arm at 3.05 (95% CI 3.00–3.10) *An. gambiae* s.l. per room per day compared with 1.9 (95% CI 1.87–1.97). There was no significant difference in sporozoite rate at 3.97% (95% CI 2.91–5.02) for the deltamethrin LLIN arm and 3.04% (95% CI 2.21–3.87) for deltamethrin + PBO LLIN arm (P = 0.17). However, when analysed by season there was some evidence that the sporozoite rate was lower in the deltamethrin + PBO LLIN arm than deltamethrin LLIN arm during the rainy/high malaria transmission seasons at 1.95% (95% CI 1.18–2.72) and 3.70% (95% CI 2.56–4.84) respectively (P = 0.01).

**Conclusions:**

While there was some evidence that *An. gambiae* s.l. sporozoite rates were lower in villages with deltamethrin + PBO LLINs during the high malaria transmission seasons of 2014–2015, there was no reduction in parity rates or indoor resting densities. There was also no evidence that permethrin + PBO LLINs provided any improved control when compared with permethrin LLINs. Combination nets may have a greater impact in areas where mixed function oxidases play a more important role in pyrethroid resistance.

## Background

Long-lasting insecticidal nets (LLINs) and indoor residual spraying (IRS) are frontline tools for malaria vector control. As a result of renewed commitment and increased funding for the control and elimination of malaria, vector control has been significantly scaled up since 2000 [1]. There is clear evidence that high coverage and utilization of LLINs reduces malaria mortality and morbidity rates and improves pregnancy outcomes in a range of transmission settings [2]. Prior to 2007, children under 5 years of age and pregnant women were the primary targets for the distribution of LLINs. A significant policy shift occurred in 2007 when the World Health Organization (WHO) issued a position statement promoting universal coverage of LLINs [3]. Since 2007 there has been a rapid increase in the distribution and ownership of LLINs in most malaria endemic countries. In sub-Saharan Africa, households owning at least one LLIN have increased from < 2% in 2000 to 55% (95% CI 50–58%) in 2015 [4]. The investment in malaria vector control including LLIN distribution and IRS appears to be justified. Between 2000 and 2015 it is estimated that *Plasmodium falciparum* infection prevalence in Africa was reduced by 50%, with LLINs and IRS contributing to 81% of this decline [5]. Vector control with LLINs and IRS is recommended by WHO as part of a national integrated vector management (IVM) plan. IVM is defined as a rational decision-making process to optimize the use of resources, promoting the use of a range of interventions, alone or in combination, selected on the basis of local evidence [6]. In Mali (West Africa), there has been particularly impressive progress in LLIN coverage. Mass nationwide distribution of LLINs and IRS in select districts have been primary elements of the national malaria control strategy in Mali since 2007. According to the 2012/13 demographic and health survey 84% of households owned at least one LLIN, while 69% of children under five and 73% of pregnant women slept under an LLIN the previous night [7]. By 2015 this had increased to 92% of households in Mali having at least one LLIN, with an average of 3 LLINs per house [8].

Despite the progress seen in Mali and across sub-Saharan Africa, there is growing concern that widespread vector resistance to pyrethroid insecticides may reduce the effectiveness of LLINs. Experimental hut trials in Benin showed a substantial reduction in the efficacy of LLINs in an area of pyrethroid resistance as long ago as 2005 [9]. More recent larger scale community trials in Benin and Senegal provided some evidence that pyrethroid resistance has reduced the effectiveness of LLINs [10, 11]. However, there are several factors that may contribute to sustained control despite high levels of resistance; such as restoration of susceptibility in older, more epidemiologically important mosquitoes, inhibition of *P. falciparum* development in resistant mosquitoes, and the physical barrier of an intact net [12, 13]. A recent multi-country study co-ordinated by WHO in Benin, Cameroon, India, Kenya and Sudan provided evidence that LLINs provided personal protection against malaria in areas with pyrethroid resistance [14].

Insecticide resistance testing in 13 sites located in southern and central Mali in 2012 demonstrated *Anopheles gambiae* sensu lato (s.l.) resistance to deltamethrin and lambda-cyhalothrin in all locations [15]. CDC bottle bioassays conducted in 2015 demonstrated high intensity of pyethroid resistance for *An. gambiae* s.l. in all 13 sites when tested with 10 times the diagnostic dose of deltamethrin and permethrin [16]. Based on the widespread and intense level of pyrethroid resistance in Mali, alternative LLIN options are being investigated to determine whether there is any advantage over mono-treated pyrethroid LLINs. At present, World Health Organization Pesticide Evaluation Scheme (WHOPES) only recommends LLINs that are treated with pyrethroid insecticides. The only alternative products are combination LLINs treated with a pyrethroid insecticide plus piperonyl-butoxide (PBO). PBO is a synergist that inhibits the activity of mixed function oxidases (MFOs) in pyrethroid resistant mosquitoes. Metabolic resistance is complex and several mixed function oxidases (MFOs) are often involved, but key enzymes responsible for pyrethroid detoxification have been repeatedly identified, such as *Cyp*6P3 and *Cyp*6M2 in *An. gambiae* [17]. Other metabolic mechanisms of insecticide resistance include esterases and glutathione-S-transferase (GST) enzymes [18]. Another common group of resistance mechanisms are target site mutations, including voltage-gated sodium channel (*Vgsc*) 1014F, *Vgsc*-1014S and *Vgsc*-1575Y [19]. PBO LLINs can restore susceptibility in mosquitoes where metabolic resistance through MFOs is the major mechanism, but have little impact on resistance caused by other mechanisms such as target site mutations. In reality, multiple mechanisms are usually involved in pyrethroid resistance in *An. gambiae* s.l. and the impact of PBO depends on the relative contribution of MFOs [19].

Deltamethrin + PBO and permethrin + PBO LLINs are designed for the control of both susceptible and pyrethroid-resistant mosquito populations through the combination of a pyrethroid with the synergist PBO. Both deltamethrin + PBO (PermaNet 3.0) and permethrin + PBO (Olyset Plus) LLINs received interim WHOPES recommendation for use as LLINs for malaria vector control in 2008 and 2012 respectively [20, 21]. Semi-field experimental hut trials of deltamethrin + PBO LLINs indicated significantly greater mortality of pyrethroid resistant *An. gambiae* s.l. than mono-treated deltamethrin LLINs in Benin, Burkina Faso and Cameroon, with the greatest increase seen with unwashed LLINs [38, 39]. While there is only one report from Benin showing that permethrin + PBO LLINs provided greater control of *An. gambiae* s.l. than permethrin LLIN [40]. While the data from experimental hut trials is promising, WHO recommended that further large-scale studies were needed to confirm their effectiveness against wild pyrethroid resistant mosquitoes and their cost-effectiveness compared with conventional LLINs. PermaNet 3.0 data was subsequently reviewed by the WHO Vector Control Advisory Group (VCAG), who supported the claim of increased efficacy against malaria vectors with cytochrome P450-based metabolic pyrethroid resistance relative to pyrethroid-only LLINs [22]. This was followed, in 2015, by the WHO Evidence Review Group (ERG) recommendation that pilot exploratory implementation be undertaken accompanied by robust evaluation [23]. This study was conducted in Southern Mali over 2 years (2014 and 2015) and compared entomology parameters between village clusters where combination LLINs were distributed compared with mono-treated pyrethroid LLINs. Entomological parameters included indoor vector resting density, vector longevity, sporozoite rates, and human blood index.

## Methods

### Selection of study sites

Based on recent insecticide resistance data the Sikasso region was selected for the combination LLIN study [15]. Testing was conducted between August and September 2013 to determine pyrethroid resistance status of *An. gambiae* s.l. and whether resistance was associated with elevated expression of MFOs. Eleven villages from Selingue district and 14 villages from Bougouni district were included for resistance testing. Mosquitoes were collected as larvae or pupae in typical breeding sites of *An. gambiae* s.l., such as temporary pools of standing water. Larvae were reared to adults in an insectary and tested by exposing four replicates of 20–25 sugar-fed adult *An. gambiae* s.l., aged 3–5 days, for 30 min in 250 ml glass bottles coated with a diagnostic dose of permethrin (21.5 µg/bottle) or deltamethrin (12.5 µg/bottle) [24]. To determine the contribution of metabolic resistance mechanisms due to mixed function oxidases, adult mosquitoes were pre-exposed to PBO (400 µg/bottle) for 1 h prior to exposure with permethrin or deltamethrin [24].

### LLIN treatments

The deltamethrin + PBO LLIN (PermaNet 3.0) is a combination of two fabrics: the roof is comprised of a knitted 100 denier monofilament polyethylene fiber blended with deltamethrin 4 g/kg (~ 180 mg ai/m^2^) + piperonyl butoxide (PBO) 25 g/kg and side panels are comprised of knitted multifilament polyester (75 denier) fibers coated with deltamethrin [20]. The side netting has two parts: a strengthened lower part, so-called border (70 cm) treated at ~ 115 mg ai/m^2^ and the rest of the side panels at ~ 85 mg ai/m^2^. The deltamethrin LLIN (PermaNet 2.0) precursor is a knitted multifilament polyester (75 denier) net coated with deltamethrin at 55 mg ai/m^2^ [20].

The permethrin + PBO LLIN (Olyset Plus) is made of mono-filament polyethylene yarn, containing 2% (w/w) technical permethrin (40:60 *cis*:*trans* isomer ratio), corresponding to 20 g ai/kg (about 800 mg ai/m^2^); and 1% (w/w) PBO, corresponding to 10 g PBO/kg (about 400 mg PBO/m^2^) [21]. Permethrin and PBO are incorporated into filaments and diffuse to the surface. The permethrin LLIN (Olyset) precursor has the same specifications minus PBO [25].

### Treatment allocation and village characteristics

**Fig. 1:**
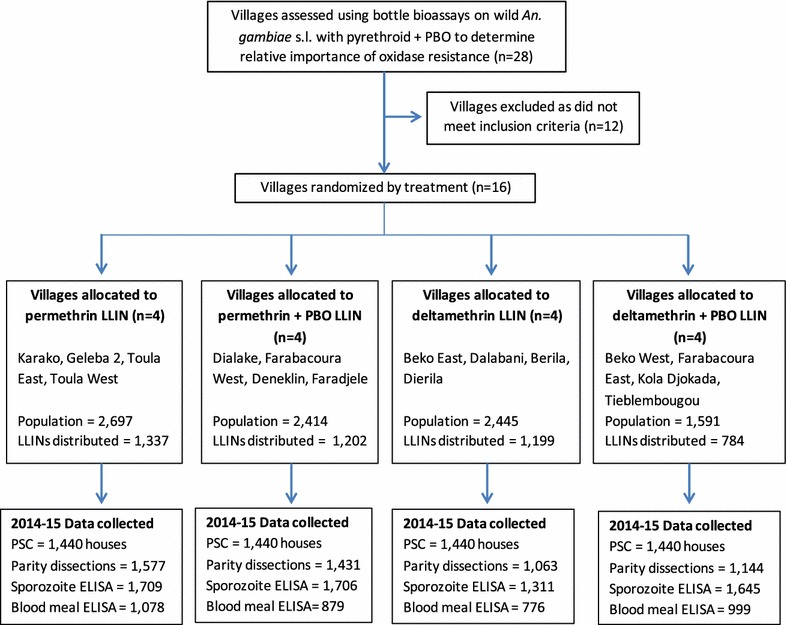
Flow chart demonstrating allocation of treatments to village clusters and data collected in 2014–2015

**Fig. 2:**
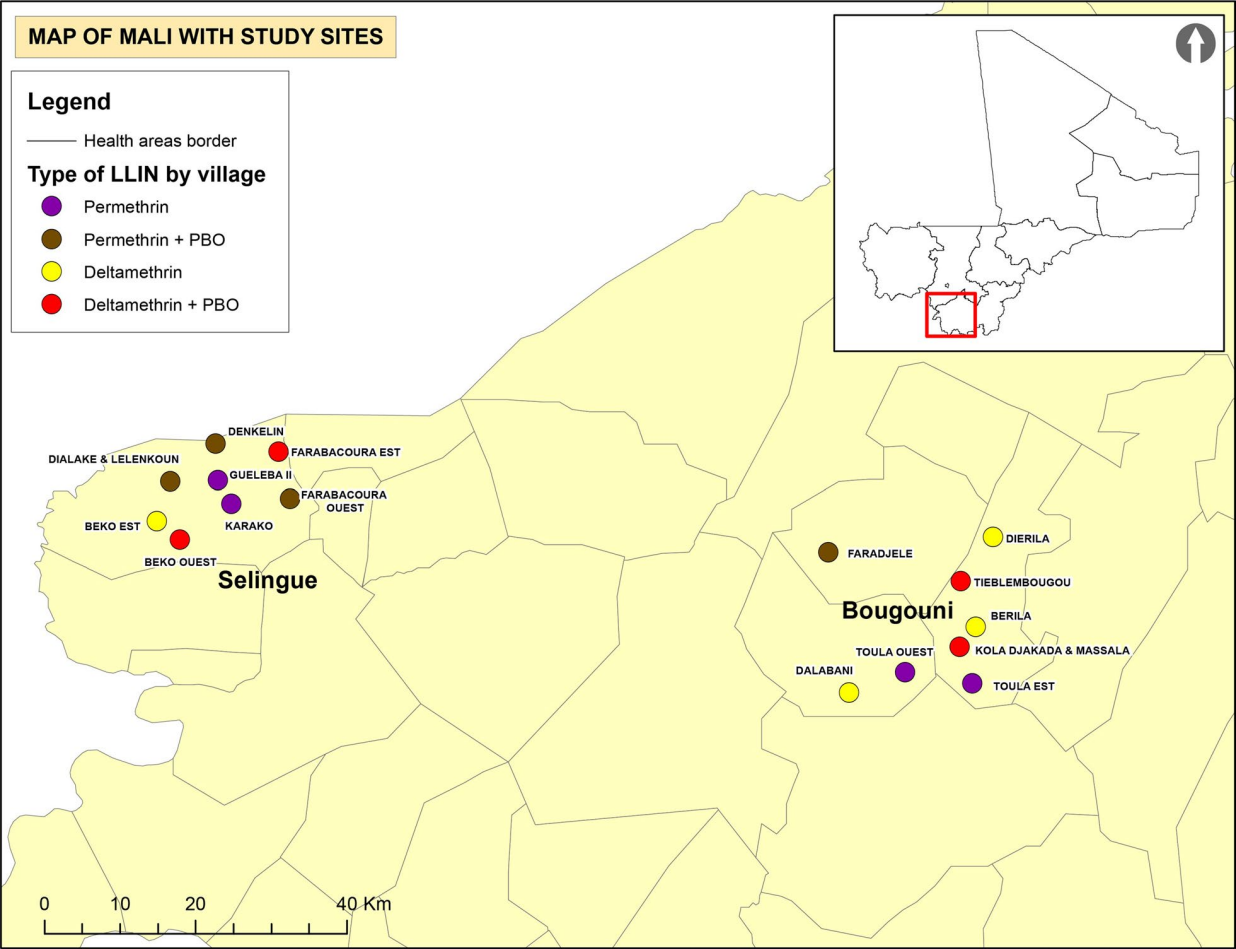
Geographical distribution of study villages in Selingue and Bougouni. Districts and treatment allocation

Twenty-five villages, with an average of 159 structures, were tested for resistance to pyrethroids and involvement of MFO resistance. Inclusion criteria were the presence of resistant *An. gambiae* s.l. and a significant increase in mortality with the addition of PBO to either permethrin or deltamethrin. Selection criteria also included population size, ease of access for vector collection during the rainy and dry seasons, sizeable vector populations during rainy seasons, and absence of indoor residual spraying (IRS). Sixteen of the villages met these criteria and were randomized to receive one of four LLIN products (four villages per arm) (Fig. 1). The altitude in the study area was similar for all villages and ranged from 200 to 350 m. Block randomization was done by assigning random numbers to each treatment. Following random assignment of treatments to each of the 16 villages, enumeration of the population (9142 persons) and structures (2546) was completed by Population Services International (PSI) in January 2014 to estimate the number of LLINs needed for each village. In total 4522 LLINs were distributed in February 2014 based on the number of residents per house to achieve universal coverage defined as 1 net for every 2 persons (Fig. 2).

### Vector sampling methods

Monitoring of indoor resting density, species composition, and longevity of vector species was conducted in all 16 villages after the distribution of LLINs. Monitoring was conducted during the rainy season (high malaria transmission) between June and November, followed by a dry season between December and May. To sample indoor resting mosquitoes, pyrethrum spray catch (PSC) and Prokopack aspirator methods were used during both years (2014 and 2015). Four teams visited the village clusters every 2 months for 16 days of sampling. A total of 30 houses were randomly selected (15 with PSC and 15 using Prokopack aspirators) for indoor vector sampling each day. In total 480 rooms were sampled during each 2 month period of the study (30 rooms per village cluster).

Adult mosquito collections were carried out indoors between 6 and 10 a.m. After laying down white sheets and closing all doors and windows, a commercial aerosol, KILIT, which consists of three different pyrethroid insecticides (d-tetramethrin 0.135% w/w, d-allethrin 0.06% w/w, cypermethrin 0.46% w/w), was sprayed. Any mosquitoes knocked-down after 10 min were put in containers and transported to the insectary for species identification using the taxonomic key of Gillies and Coetzee [26]. All unfed and freshly fed mosquitoes were dissected to determine parity rates according to the ovary tracheation method described by Detinova [27] (Fig. 1). Presence of tracheole skeins was used to classify mosquitoes as nulliparous or parous. A subsample of collected mosquitoes was preserved individually in Eppendorf tubes with silica gel for subsequent molecular analysis.

Baseline entomology data was collected by indoor PSC collections during the dry season in December 2013 in approximately 50 houses per treatment arm, prior to distribution of LLINs. ANOVA analysis indicated that there was no difference in baseline indoor resting densities for villages located in Selingue or Bougoni districts when analysed according to treatment allocation.

### Laboratory mosquito analysis

Subsamples of *An. gambiae* s.l. collected using PSC and Prokopack aspirator were tested to determine *P. falciparum* circumsporozoite rates using ELISA [28]. All freshly fed and half gravid *An. gambiae* s.l. were analysed by ELISA to determine the mammalian host blood meal origin using antigen to detect human or bovine hosts [29]. A subsample of *An. gambiae* s.l. collected resting indoors were analysed for species identification using the method of Scott et al. [30]. This was used to distinguish between *Anopheles arabiensis* and *An. gambiae* sensu stricto (s.s.).

### Data analysis

#### Indoor resting density

PSC and Prokopack data were used to calculate the mean density of vectors in a room per village cluster using the formula: Number of vectors collected/total number of rooms surveyed [28]. The number of *An. gambiae* s.l. collected was compared among the different treatments and village clusters using the negative binomial regression Z test for differences in proportions using Epi6 (Epi Info version 6, CDC, Atlanta, USA).

#### Parity

The parity rate of morphologically identified *An. gambiae* s.l. was calculated using the formula: Total number of vectors parous/number of vectors dissected * 100 [28]. The Chi square test was used to compare parity rates.

#### Mortality

Percent mortality for bioassays was calculated as: total number of mosquitoes unable to fly (dead) after 30 min exposure/total number of mosquitoes tested * 100. When control mortality was between 5 and 20%, observed mortality was corrected using Abbott’s formula. Experiments were repeated when control mortality was greater than 20%. The Chi square test was used to compare mortality.

#### Sporozoite rate

The sporozoite rate of identified *An. gambiae* s.l. was calculated using the formula:

Total number of vectors positive with *P. falciparum* sporozoite/number of vectors tested * 100 [28]. The Chi square test was used to compare sporozoite rates.

#### Human blood meal index

The human blood index (HBI) of identified *An. gambiae* s.l. was calculated using the formula: total number of vectors positive with human blood meal/number of vectors tested * 100. The Chi square test was used to compare HBI.

## Results

### Resistance frequency of *Anopheles gambiae* and evidence for metabolic resistance

Results of bottle bioassays are presented for 8 villages of Selingue district and 8 villages of Bougouni district for susceptibility to permethrin and deltamethrin with and without exposure to PBO. Results are presented with village clusters grouped by the insecticide active ingredient (permethrin or deltamethrin) used on the LLIN that was later distributed (Tables 1, 2). In villages where permethrin or permethrin + PBO nets were to be distributed, mortality to permethrin in bottle bioassays was extremely low, with a mean mortality of < 2%. With pre-exposure of PBO, mortality increased significantly, but was still only 23% (95% CI 18–28) in villages allocated permethrin LLINs and 27% (95% CI 23–31) for villages allocated permethrin + PBO LLINs (Table 1). *Anopheles gambiae* s.l. were more susceptible to deltamethrin with mean mortality of 29% (95% CI 25–33) in villages allocated deltamethrin LLINs and 38% (95% CI 33–43) in those allocated deltamethrin + PBO LLINs. This increased to 66% (95% CI 61–71) and 60% (95% CI 55–65), respectively, following pre-exposure of PBO, indicating the importance of oxidase mechanisms, but that other resistance mechanisms were also involved (Table 2).

**Table 1:** Mortality of *Anopheles gambiae* s.l. following exposure to permethrin with and without pre-exposure to the synergist PBO (2013)

Village	Number tested permethrin	Number tested permethrin + PBO	% mortality permethrin (95% CI)	% mortality perm + PBO (95% CI)	P value
Permethrin LLIN distributed					
Karako	100	70	1 (< 1–5)	21 (14–34)	0.001
Geleba 2	103	103	1 (< 1–5)	43 (33–53)	0.001
Toula (east and west)^a^	102	101	1 (< 1–5)	4 (2–10)	0.175
Total	305	274	1 (< 1–3)	23 (18–29)	0.001
Permethrin + PBO LLIN distributed					
Dialake^b^	104	100	3 (< 1–8)	11 (6–19)	0.022
Lelenkou^b^	100	99	0 (0–4)	15 (9–24)	0.001
Farabacoura west	104	102	0 (0–3)	7 (3–14)	0.008
Deneklin	103	100	4 (< 1–9)	75 (65–83)	0.001
Faradjele^c^	Not tested				n/a
Total	411	401	2 (1–3)	27 (23–31)	0.001

^a^Toula east and west were subsequently separated into two village clusters prior to treatment allocation

^b^Due to the small size and proximity of Dialake and Lelenkou, they were subsequently included in the study as a single village cluster

^c^Due to the availability of mosquitoes only deltamethrin and deltamethrin + PBO were tested in Faradjele. The mortality was 38% (n = 99) with deltamethrin and 77% (n = 103) with deltamethrin + PBO. The result showed a significant increase of mortality after PBO exposure (P = 0.0001)

**Table 2:** Mortality of *Anopheles gambiae* s.l. following exposure to deltamethrin with and without pre-exposure to the synergist PBO (2013)

Village	Number tested deltamethrin	Number tested deltamethrin + PBO	% mortality deltamethrin (95% CI)	% mortality delta + PBO (95% CI)	P value
Deltamethrin LLIN distributed					
Beko east	103	69	0 (0)	18 (10–30)	0.001
Dalabani	101	102	50 (40–61)	71 (61–79)	0.003
Berila	104	101	13 (7–20)	81 (72–88)	0.001
Dierila	102	103	57 (47–67)	76 (66–84)	0.004
Total	410	375	29 (24–33)	66 (61–71)	0.001
Deltamethrin + PBO LLIN distributed					
Beko west	103	69	0 (0)	17 (10–30)	0.001
Farabacoura east	101	103	88 (80–94)	92 (85–97)	0.323
Kola Djakada	104	99	20 (13–29)	47 (37–58)	0.001
Tieblembougou	100	102	47 (37–57)	66 (56–75)	0.007
Total	408	373	38 (33–33)	60 (55–65)	0.001

### Vector species composition

The predominant species complex present throughout the study was *An. gambiae* s.l. PCR using the method of Scott et al. indicated that *An. arabiensis* was found at very low frequency resting indoors (< 1%), throughout both the rainy and dry seasons of 2014 and 2015. No subsequent PCR method was conducted to differentiate between *An. gambiae* s.s. and *Anopheles coluzzii* and, therefore, the mosquitoes are subsequently referred to as *An. gambiae* s.l. [30].

### Impact of LLIN treatments on *An. gambiae* indoor resting densities, sporozoite rates, parity rates and blood meal host

Baseline PSC collections in December 2013 produced a mean density of 1.06 (95% CI 0.78–1.34) and 1.51 (95% CI 1.27–1.75) *An. gambiae* s.l. per room per day for the permethrin and permethrin + PBO LLIN arms respectively. For the deltamethrin LLIN and deltamethrin + PBO LLIN arms, resting densities were 0.25 (95% CI 0.01–0.53) and 2.51 (95% CI 2.23–2.79) per room per day. The sporozoite rates were 8.9% (95% CI 0.6–17.2) and 1.4% (95% CI 0.1–4.2) for the permethrin and permethrin + PBO LLIN arms respectively. For the deltamethrin LLIN and deltamethrin + PBO LLIN arms the sporozoite rates were 0 and 8.9% (95% CI 1.5–16.4). Baseline mosquito trapping gave some indication that study villages may not have been equivalent before intervention, particularly the resting density for the deltamethrin LLIN arms, although the baseline was limited to 1 month due to the short rainy season in Mali.

#### Permethrin LLIN vs permethrin + PBO LLIN arms

Indoor mosquito resting densities were significantly different for permethrin and permethrin + PBO treatment arms during 2014–2015 at 3.21 (95% CI 3.16–3.26) and 3.70 (95% CI 3.65–3.76) *An. gambiae* s.l. captured per room/day (P = 0.001) (Table 3). The sporozoite rate was similar at 5.42% (95% CI 4.37–6.46) in the permethrin LLIN arm and 6.92% (95% CI 5.71–8.12) for permethrin + PBO LLIN during 2014/15 (P = 0.06) (Table 3). However, the sporozoite rate was significantly higher in the permethrin + PBO LLIN arm at 11.60% (95% CI 8.49–14.72) compared to 6.90% (95% CI 4.12–9.68) for the permethrin LLIN arm (P = 0.03) during the dry seasons (Table 4). There was no difference in parity rates over the 2 years of the trial (Table 3). The human blood index was slightly lower in the permethrin + PBO LLIN arm at 46.3% (95% CI 43.0–49.6) than the permethrin LLIN arm 55.9% (95% CI 53.0–58.9) (P = 0.001) during 2014/15 (Table 3). Overall for all sites, 28.7% (95% CI 27.2–30.2) of samples were not reactive in blood-meal ELISA tests, with the remainder (15–25%) being bovine-fed.

**Table 3:** Indoor resting density, parity rate, sporozoite and human blood meal index rate of *Anopheles gambiae* s.l. collected from village clusters with each LLIN intervention 2014/15

LLIN treatment arm	Total resting indoors	Collection houses	*An. gambiae* s.l. per room per day (95% CI)	P value
Indoor resting density of *An. gambiae* s.l.				
Permethrin	4624	1440	3.21 (3.16–3.26)	0.001
Permethrin + PBO	5335	1440	3.70 (3.65–3.76)	
Deltamethrin	2766	1440	1.92 (1.87–1.97)	0.001
Deltamethrin + PBO	4385	1439	3.05 (3.00–3.10)	

LLIN treatment arm	Number dissected	Number parous	Parity rate % (95% CI)	P value
Parity rate of indoor resting *An. gambiae* s.l.				
Permethrin	1577	1242	78.8 (76.7–80.8)	0.08
Permethrin + PBO	1431	1089	76.1 (73.9–78.3)	
Deltamethrin	1063	837	78.7 (76.3–81.2)	0.05
Deltamethrin + PBO	1144	862	75.4 (72.9–77.9)	

LLIN treatment arm	Number tested	Number positive sporozoite	% sporozoite positive	P value
Sporozoite rate of indoor resting *An. gambiae* s.l.				
Permethrin	1809	98	5.42 (4.37–6.46)	0.06
Permethrin + PBO	1706	118	6.92 (5.71–8.12)	
Deltamethrin	1311	52	3.97 (2.91–5.02)	0.17
Deltamethrin + PBO	1645	50	3.04 (2.21–3.87)	

LLIN treatment arm	Number tested	Number positive human	% HBI (95% CI)	P value
Human blood meal index (HBI) of indoor resting *An. gambiae* s.l.				
Permethrin	1078	603	55.9 (53.0–58.9)	0.001
Permethrin + PBO	879	407	46.3 (43.0–49.6)	
Deltamethrin	771	397	51.5 (48.0–55.0)	0.79
Deltamethrin + PBO	883	449	50.8 (47.6–54.1)

**Table 4:** Indoor resting density, parity rate, sporozoite rate and human blood meal index of Anopheles gambiae s.l. separated into dry (February, April, December) and rainy (June, August, October) seasons collected from village clusters with each LLIN intervention 2014/15

Dry season					Rainy season			
Treatment	Total resting indoors	Collection days	Resting density/room/day (95% CI)	P value	Total resting indoors	Collection days	Mosquito/room/day (95% CI)	P value
Indoor resting density of *An. gambiae* s.l.								
Permethrin	866	720	1.20 (1.13–1.28)	0.001	3758	720	5.22 (5.15–5.29)	0.001
Permethrin + PBO	1452	720	2.02 (1.94–2.09)		3883	720	5.39 (5.32–5.47)	
Deltamethrin	391	720	0.54 (0.47–0.62)	0.001	2375	720	3.30 (3.23–3.37)	0.001
Deltamethrin + PBO	981	719	1.36 (1.29–1.44)		3404	720	4.73 (4.66–4.80)	

Dry season					Rainy season			
Treatment	Number dissected	Number parous	Parity rate (95% CI)	P value	Number dissected	Number parous	Parity rate (95% CI)	P value
Parity rate of *An. gambiae* s.l.								
Permethrin	370	300	81.1 (77.1–85.1)	0.15	1207	942	78.0 (75.7–80.4)	0.18
Permethrin + PBO	480	370	77.1 (73.3–80.8)		951	719	75.6 (72.9–78.3)	
Deltamethrin	178	162	91.0 (86.8–95.2)	0.01	885	676	76.4 (73.6–79.2)	0.06
Deltamethrin + PBO net	321	265	82.6 (78.4–86.7)		823	597	72.5 (69.5–75.6)	

Dry season					Rainy season			
Treatment	Number tested	Number of SPZ+	% sporozoite positive (95% CI)	P value	Number tested	Number of SPZ+	% sporozoite positive (95% CI)	P value
Sporozoite rate of *An. gambiae* s.l.								
Permethrin	319	22	6.90 (4.12–9.68)	0.03	1490	76	5.10 (3.98–6.22)	0.67
Permethrin + PBO	405	47	11.60 (8.49–14.72)		1301	71	5.46 (4.22–6.69)	
Deltamethrin	257	13	5.06 (2.38–7.74)	0.50	1054	39	3.70 (2.56–4.84)	0.01
Deltamethrin + PBO	413	26	6.30 (3.95–8.64)		1232	24	1.95 (1.18–2.72)	

Dry season					Rainy season			
LLIN treatment arm	Number tested	Number human positive	% HBI (95% CI)	P value	Number tested	Number positive HBI	% HBI (95% CI)	P value
Human blood meal index of indoor resting *An. gambiae* s.l.								
Permethrin	258	124	48.1 (42.0–54.2)	0.17	820	479	58.4 (55.0–61.8)	< 0.01
Permethrin + PBO	289	122	42.2 (36.5–47.9)		590	285	48.3 (44.3–52.3)	
Deltamethrin	186	101	54.3 (47.1–61.5)	0.44	585	296	50.6 (46.5–54.6)	0.195
Deltamethrin + PBO	327	189	57.8 (52.4–63.2)		556	260	46.8 (42.6–50.9)	

#### Deltamethrin vs deltamethrin + PBO arms

There was a consistently higher density of *An. gambiae* s.l. collected in the deltamethrin + PBO LLIN treatment arm at 3.05 (95% CI 3.00–3.10) than the deltamethrin LLIN arm at 1.92 (95% CI 1.87–1.97) throughout 2014–2015 (P = 0.001) (Table 3). The sporozoite rate was similar at 3.97% (95% CI 2.91–5.02) in the deltamethrin LLIN arm and 3.04% (95% CI 2.21–3.87) for deltamethrin + PBO arm during 2014/15 (P = 0.17) (Table 3). However, when broken down to dry and rainy seasons over 2 years, the sporozoite rate was significantly lower in the deltamethrin + PBO net arm at 1.95% (95% CI 1.18–2.72) compared to 3.70% (95% CI 2.56–4.84) for the deltamethrin LLIN (P = 0.01) during the rainy seasons (Table 4). There was no difference in parity rates during the rainy seasons over the 2 years of the trial (Table 3). The human blood index was around 50% for both deltamethrin LLIN arms (P = 0.79), with a similar proportion of non-reactive and bovine-fed specimens as in the permethrin LLIN arms.

## Discussion

*Anopheles gambiae* s.l. was the predominant species present in all villages over the duration of the 2-year trial. The frequency of *An. gambiae* s.l. resistance to permethrin and deltamethrin was very high among the study villages before the distribution of LLINs for the trial. The impact of PBO pre-exposure in bottle bioassays did not fully restore susceptibility for either insecticide and in some villages the increase in mortality was minimal. There was a much greater increase in mortality for deltamethrin than permethrin in bottle bioassays following PBO pre-exposure, which indicated an important role of oxidase-based resistance, although other mechanisms were likely involved as well. Based on this evidence of oxidase-based pyrethroid resistance, the hypothesis was that LLINs containing PBO would kill a greater proportion of malaria vectors than the respective pyrethroid only mono-treatments. Combination LLINs were predicted to have a substantial impact on the vectorial capacity by reducing the number of mosquitoes that survive the parasites intrinsic incubation period (monitored by parity and sporozoite rates) and by reducing the human biting rate (monitored by PSC) [31]. Indoor resting densities were used as a proxy for human biting rate due to the difficulties associated with conducting human landing catches on a large scale. The use of resting densities as a proxy for biting rates is described by WHO and has been used in several trials to determine the impact of interventions [32, 33]. This is considered a suitable proxy for endophilic species where few blood-fed mosquitoes are likely to exit before conducting PSC [33]. However, it is a study limitation that data was not collected on actively host-seeking mosquitoes and that no data was collected using outdoor sampling methods. Contrary to the study hypothesis, resting densities were significantly greater for the deltamethrin + PBO LLIN arm than deltamethrin LLIN arm. Deltamethrin + PBO LLINs have a greater dose of deltamethrin and also PBO on the roof of the net, but neither of these factors explains the apparent reduced impact on resting densities compared to the deltamethrin LLIN arm. Baseline mosquito trapping conducted for 1 month prior to distribution of LLINs gave some indication that study villages may not have been equivalent before intervention; with a mean resting density of 0.25 (0.01–0.53) *An. gambiae* s.l. per room per day in the deltamethrin LLIN arm compared to 2.51 (2.23–2.79) in the deltamethrin + PBO LLIN arm (although the baseline period was limited due to the short rainy season in Mali).

When analysed over the 2 year duration of the study there was no evidence of any difference in sporozoite rate between the respective combination LLINs and pyrethroid mono-treatments. However, when analysed by season there was evidence that villages with deltamethrin + PBO LLINs had a lower sporozoite rate than those with deltamethrin LLINs during the rainy seasons. In this study LLIN usage was not monitored, however in a 2015 Malaria Indicator Survey the nationwide ratio of use to access was > 90%, with 71% of Sikasso Region (where the study was located) reporting sleeping under an ITN the previous night [34]. In some regions of Mali it is common for people to sleep outdoors without mosquito nets either for part of or all night during the dry season to avoid the hot and stifling conditions indoors [35, 36]. Despite evidence that deltamethrin + PBO LLINs reduced sporozoite rates over the 2014/15 rainy seasons, there was no sizeable reduction in vector longevity as measured by parity rates (Table 4).

The *An. gambiae* s.l. human blood-feeding index was surprisingly low in all arms at between 46 and 56%. Blood-meal host preference was monitored primarily to determine whether use of combination LLINs resulted in any diversion of vectors to feed on non-human hosts. Despite their being a significant difference in the human blood index between the permethrin LLIN arms the difference was small and is unlikely to indicate any sizeable shift in feeding behaviour. Overall 29% of samples failed to react, which could be due to the insensitivity of ELISA for specimens where blood-meals have been partially digested, or may indicate that mosquitoes fed on other animals which weren’t tested, such as goats, sheep or donkeys. *Anopheles gambiae* s.s. is generally regarded as an anthropophilic species, however, in western Kenya the human blood index was 53%, with a large proportion having fed on livestock including cattle and goats [37]. The relatively low human blood index in both cases may be due to the high coverage of LLINs and close proximity of livestock resulting in opportunistic feeding patterns.

The sporozoite rates for permethrin LLINs, at 5.4 and 6.9% (mono-treated and combination), were significantly greater than for deltamethrin LLINs, at 3.0 and 3.9%. In bottle bioassays the frequency of resistance was far greater for permethrin than deltamethrin and this finding of higher sporozoite rates in areas where permethrin LLINs were used may be a sign of partial control failure for permethrin LLINs.

Semi-field experimental hut trials of deltamethrin + PBO LLINs have indicated significantly greater mortality of pyrethroid resistant *An. gambiae* s.l. than mono-treated deltamethrin LLINs in both Benin, Burkina Faso and Cameroon, with the greatest increase seen with unwashed LLINs [38, 39]. While there is only one report from Benin showing that permethrin + PBO LLIN provided greater control of *An. gambiae* s.l. than permethrin LLIN [40]. Further experimental trials in India and Tanzania were conducted against susceptible *Anopheles* as part of the WHOPES evaluation process but provide no evidence to indicate any improvement of combination LLINs over mono-treated LLINs.

The only published village scale study was in Nigeria where a 12 month village level trial appeared to produce greater impact on vector resting density, sporozoite rates and parity in a village with deltamethrin + PBO LLINs than deltamethrin LLINs in an area of *An. gambiae* with pyrethroid resistance attributed to both *Vgsc*-1014F and MFOs [41]. However, this was a particularly small study with 1 village per arm and no baseline data. To date, this is the largest village level trial to assess the performance of combination LLINs.

## Conclusion

Over the 2 years of the trial neither deltamethrin + PBO LLINs nor permethrin + PBO LLINs provided a meaningful improvement over deltamethrin or permethrin LLINs, respectively. It is important to recognize that during village selection, bottle bioassays with pre-exposure to PBO resulted in improved mortality but did not restore vector susceptibility; particularly for permethrin the increase in mortality was relatively small. LLINs containing PBO may have a greater impact in areas where mixed function oxidases play a more important role in pyrethroid resistance.
